# Genetic structure analysis and identifying key founder inbred lines in diverse elite sub-tropical maize inbred lines

**DOI:** 10.1038/s41598-023-38980-3

**Published:** 2023-07-20

**Authors:** Melaku Gedil, Abdoul-Raouf Sayadi Maazou, Degife A. Zebire, Ana Luísa Garcia-Oliveira, Nnanna Unachukwu, César Petroli, Sarah Hearne, Leslie A. Everett, Soon-Kwon Kim, Abebe Menkir

**Affiliations:** 1 International Institute of Tropical Agriculture, PMB 5320, Ibadan, 200001 Nigeria; 2 Kindo Seeds, Niamey, Niger; 3 Department of Plant Science, College of Agricultural Sciences, Arba Minch University, Arba Minch, Ethiopia; 4 International Maize and Wheat Improvement Center, Excellence in Breeding, Ibadan, Nigeria; 5 International Maize and Wheat Improvement Center, Carretera México-Veracruz Km. 45 El Batán, Texcoco, C.P. 56237 México; 6 International Maize and Wheat Improvement Center (CIMMYT), ICRAF House, UN Avenue, Nairobi, P.O. Box 1041-00621, Kenya; 7 Department of Molecular Biology, College of Biotechnology, CCS Haryana Agricultural University, Hisar, 125004 Haryana India; 8 Department of Agronomy and Plant Genetics, University of Minnesota, 411 Borlaug Hall, 1991 Upper Buford Circle, Saint Paul, MN 55108 USA; 9 Handong Global University, Pohang, Republic of Korea; 10 International Corn Foundation, Pohang, Republic of Korea

**Keywords:** Plant breeding, Plant breeding, Plant genetics

## Abstract

Understanding the genetic relationships between the key founder inbred lines and derived inbred lines could provide insight into the breeding history and the structure of genetic diversity of the available elite inbred lines with desirable target traits. The maize improvement program at the International Institute of Tropical Agriculture (IITA) analyzed the pedigree information of 623 sub-tropical maize inbred lines generated at the IITA maize breeding program to identify the key founder inbred lines. We also used 5032 SNP markers to assess the genetic similarities of the founder inbred lines with their progenies subsequently developed for specific target traits. The results of pedigree analysis and SNP markers-based similarity scores identified 20 key founder inbred lines with significant contributions to the development of drought tolerant, early maturing, productive, *Striga* resistant, provitamin A enriched, and quality protein maize inbred lines. In our breeding program, line TZMi501 belonging to a flint heterotic group (HGA), and TZMi407-S and TZMi214, representing the dent heterotic group (HGB), were identified as the most useful founder inbred lines. The 623 inbred lines were consistently separated into four clusters based on Ward’s hierarchical clustering, structure, and principal component analyses, with the 20 founder inbred lines spread into all clusters. The founder inbred lines were more genetically related to the productive inbred lines but showed genetic divergence from the provitamin A enriched inbred lines. These results provide a better understanding of the breeding history of the sub-tropical maize inbred lines to facilitate parental selection aligned to existing heterotic groups for use in breeding programs targeting the improvement of essential traits in maize.

## Introduction

Maize has emerged as a major staple food crop for millions of people in West and Central Africa (WCA). The crop is cultivated in the lowland tropics and sub-tropics that differ in seasonal rainfall, temperatures, elevation, and length of the growing period that affect the presence of different types of diseases, pests, and other adaptive traits^1^. The maize breeding program at the International Institute of Tropical Agriculture^2^ has thus made a modest investment in the development of germplasm required for optimal response to sub-tropical production environments in Nigeria, Cameroon, and other countries in WCA^2^. The breeding program has focused on improving key traits that affect production, meet consumer needs, and confer consistent performance across environments^3^.

The breeding programs in Nigeria developed a sub-tropical source population known as TZMSR from crosses of maize varieties and hybrids from Eastern, Southern, and Central Africa, as well as germplasm from the International Maize and Wheat Improvement Center (CIMMYT) and the temperate zone with tropical maize streak virus (MSV) resistant maize germplasm^2^. Additional sub-tropical populations were formed in Cameroon by crossing TZMSR to hybrids from Kenya, Zimbabwe, DEKALB Seed Company, and diverse germplasm from CIMMYT, Tanzania, Kenya, Costa Rica, Guatemala, Brazil, and Thailand. The diverse sub-tropical populations developed in Nigeria and Cameroon had been sources of elite maize inbred lines for developing high-yielding hybrids with resistance to *turcicum* leaf blight, common rust, grey leaf spot, and the maize streak virus disease for commercialization in the two countries^2,4–7^. The most diverse elite maize inbred lines developed in Nigeria and Cameroon prior to 1995 were crossed with each other as well as with introduced and tropical inbred lines as donors of novel alleles to generate source populations for the continual development of elite maize inbred lines with desirable agronomic features, tolerance to drought, resistance to *Striga hermonthica*, earliness, high lysine, tryptophan, and provitamin A content for two decades. Understanding the contributions of pre-1995 elite inbred lines to the subsequent development of elite inbred lines with different target improved traits can thus help in identifying vital inbred lines with significant impact in our breeding program. A thorough understanding of the founder inbred lines may also provide insights into the consequences of the breeding strategy used in managing the genetic diversity of the sub-tropical maize germplasm in IITA^8^.

Effective use of existing genetic diversity is a critical prerequisite for achieving sustained genetic gain in productivity, stress resilience, and improved nutritional quality. A comprehensive analysis of pre-1995 elite sub-tropical maize inbred linesin the continual development of inbred lines over the years is vital in identifying key maize inbred lines contributing the most to the breeding program. Likewise, such analyses can help select underutilized inbred lines to broaden and diversify the genetic base of adapted sub-tropical germplasm and provide the basis for monitoring genetic diversity^8,9^. Many studies in maize used pedigree-based relationships to identify key founder inbred lines of germplasm adapted to central Europe and the United States and provided insights into the industry germplasm structure and relationships between pre-commercial and commercial maize inbred lines^8–13^. Single nucleotide polymorphism (SNP) markers have successfully confirmed the importance of the founder inbred lines in ex-PVP germplasm and identified common heterotic groups in temperate maize. Further high-density haplotype analysis using SNPs revealed significant haplotype sharing between historical founders and ex-PVP inbred lines^14^. Additionally, Minamikawa et al.^15^, using SNPs, traced founder haplotypes of Japanese apple varieties for use in genetic improvement of fruit quality traits and understanding the selection history of germplasm in their breeding program.

The present study, examined a comprehensive list of 88 elite maize parental inbred lines and their 535 elite inbred progenies adapted to sub-tropical environments. We run both pedigree and high-density SNP data-based analyses to define the genetic structure of these inbred lines and examine the contribution of the pre-1995 parental inbred lines to subsequent line development. The objectives of this study were then to (i) identify vital founder inbred lines with significant contributions to the development of sub-tropical inbred lines, (ii) assess the relationship of the founder inbred lines with the sub-tropical inbred lines, and (iii) evaluate the genetic structure and diversity of the sub-tropical inbred lines.

## Materials and methods

### Sub-tropical maize inbred lines and their ancestors

The sub-tropical elite maize inbred lines used in the present study consisted of (i) 89 drought tolerant inbred lines (DT), (ii) 11 early inbred lines (EARLY), (iii) 88 key parental inbred lines (Founder), (iv) 124 productive inbred lines (PT), (v) 86 provitamin A enriched inbred lines (PVA), (vi) 31 quality protein maize (QPM) inbred lines and (vii) 194 Striga tolerant inbred lines (STR). All of the sub-tropical inbred lines in this study have been derived from broad-based populations, varieties, biparental crosses, backcrosses, and multi-parent synthetics through repeated self-pollination with selection for desirable agronomic traits and resistance to the major diseases and other target traits. The inbred lines have undergone seven to nine generations of selfing and represent the present elite sub-tropical inbred lines in the IITA maize breeding program. A detailed summary of the genetic composition of these inbred lines and their heterotic affinities are presented in Table 1 and Supplementary Table S1.

**Table 1:** Summary of the genetic backgrounds of the inbred lines improved for target traits and the key parents used in the breeding program.

Target trait improvement	Number of inbred lines	Genetic composition
Drought tolerance (DT)	89	Consist of two adapted drought tolerant inbred lines from the DTPL-W C7 population, two inbred lines derived from backcrosses involving temperate germplasm and non-recurrent parents, and an elite tropical line (TZI3) as a non-recurrent parent, 20 inbred lines derived from bi-parental crosses of elite sub-tropical inbred lines containing MO17 in their backgrounds and adapted drought tolerant inbred lines derived from LaPostaSeq C7, and 65 inbred lines derived from biparental crosses between elite sub-tropical inbred lines and adapted drought tolerant inbred lines derived from LaPostaSeq C7
Early inbred lines (Early)	11	Adapted inbred lines derived mainly from early maturing maize varieties, namely HP97TZECOMP3 and Early-W-SR
Key parental lines inbred (Founder)	88	Composed of 62 key sub-tropical inbred lines derived from adapted variety (TZMSR), populations (POP43 SR, Coca SR), as well as crosses of TZMSR, SYNA1, and SYNB1 with inbred lines in Cameroon under the NCRE project^16^ and 26 key inbred lines derived from TZMSR, diverse source germplasm from eastern and southern Africa, and crosses of TZMSR with diverse germplasm^17^. These lines were developed before the mid-1990s and used as major sources of adaptation to sub-tropical elite inbred lines developed in subsequent years
Productive elite inbred lines	124	Composed of inbred lines derived from bi-parental crosses of the key breeding inbred lines from Cameroon and Nigeria
Provitamin A-enriched inbred lines (PVA)	86	Composed of inbred lines derived from bi-parental crosses of the key white breeding inbred lines from Cameroon and Nigeria with orange tropical provitamin A-enriched maize inbred lines
QPM inbred lines	31	Composed of inbred lines derived from bi-parental crosses of the key breeding inbred lines from Cameroon and Nigeria with CIMMYT QPM inbred lines (GQL5, CML144, CML159, and CML176)
Striga tolerant inbred lines (STR)	194	Composed of inbred lines derived from bi-parental crosses of the key breeding inbred lines from Cameroon and Nigeria with Striga tolerant/resistant tropical and sub-tropical maize inbred lines

### DNA extraction and genotyping

### DArTseq genotyping

Genotyping was performed using the high throughput, next-generation sequencing (NGS) based technology at Diversity Arrays Technology Pty Ltd (diversityarrays.com, Canberra, Australia) as described earlier^18^. From the set of 635 maize inbred lines, a total of 38,052 SNP-based DArTseq markers were generated. Molecular markers were subjected to a filtering process using TASSEL 5.2.48 (Bradbury et al.^19^); all those SNPs with a missing rate higher than 10%, minor allele frequency (MAF) < 0.05, and maximum allele frequency > 0.95 excluded from the genotypic dataset. Finally, 5032 high-quality SNPs distributed across the ten chromosomes of the maize genome were retained for subsequent analysis (Supplementary Table S2, Supplementary Figure S1).

### Pedigree and SNP-based structure and diversity analyses

The pedigree relationships between the inbred lines were analysed and visualized using Helium software version 1.19.09.03^20^ to identify the founder inbred lines with significant contributions to the development of the sub-tropical inbred lines. The similarity of the pre-1995 founder inbred lines to subsequent sub-tropical elite maize inbred lines development was also assessed based on the SNP markers using Flapjack software version 1.21.02.04^21^. The essential parameters of diversity, including gene diversity, heterozygosity, major allele frequency (MAF), and polymorphic information content (PIC), were estimated using PowerMarker version 3.25^22^. Analysis of molecular variance (AMOVA) among groups, among inbred lines within groups, and among all inbred lines was conducted using GenAlEx version 6.503^23^. A Fixation index^24^ was also calculated to determine the genetic differentiation between groups^25^.

To determine the inherent population structure of the 623 inbred lines, a model-based clustering method was implemented using Structure software version 2.3.4^26^. The simulation was performed without prior information on the individuals with the number of expected sub-populations or clusters (K) ranging from 1 to 10 with ten replications. Each replication was run with iterations and burn-ins set to 10,000. The Evanno transformation^27^ was used to determine the most appropriate K-value within the set of inbred lines using Structure Harvester (http://taylor0.biology.ucla.edu/structureHarvester/, accessed on 25 August 2022). A threshold value of ≥ 60% was used to assign each inbred line to a specific group, while the inbred lines presenting values below the threshold limit were assigned to the mixed group.

PLINK^28^ was used to calculate the pairwise genetic distance (identity-by-state, IBS) among the inbred lines for hierarchical clustering. The IBS matrix was then used to generate Ward’s minimum variance hierarchical cluster dendrogram and a neighbor-joining tree using the Analyses of Phylogenetics and Evolution (ape) package^29^ implemented in R version 4.2.0^30^. Principal Component Analysis (PCA) was also carried out in Tassel version 5.2.83^19^ to visualize the pattern of genetic dissimilarities within and between sub-groups.

### Ethical approval and consent to participate

The use of plant material complies with relevant institutional, national, and international guidelines and legislation.

## Results

### Identification of key founder inbred lines

**Figure 1:**
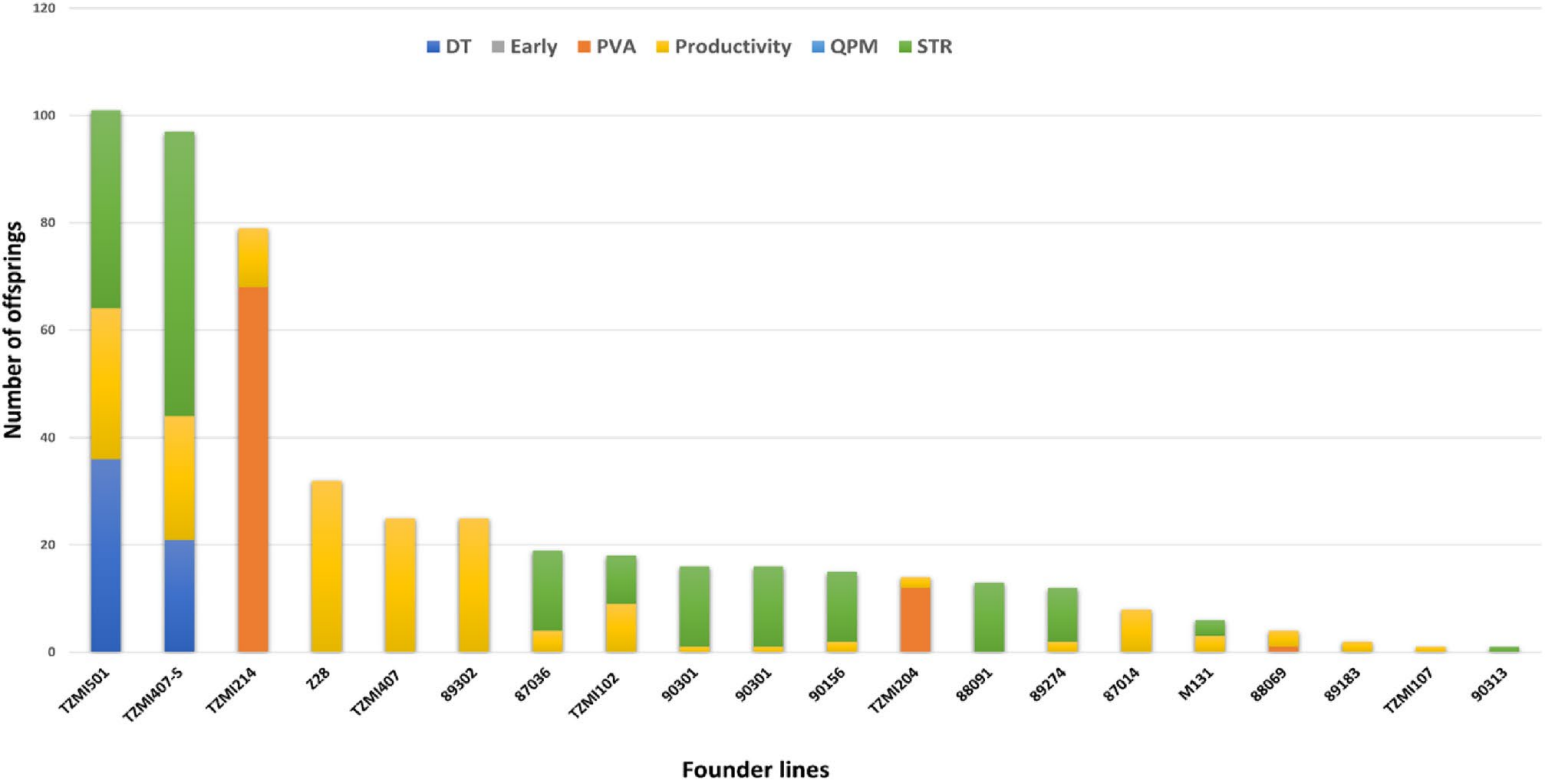
Number of offsprings obtained from 20 key founder inbred lines identified through pedigree analysis. Each color indicate a purpose of development of the offsprings. *PVA *Provitamin A, *STR Striga* resistance, *DT *Drought tolerance, *Early *Early maturity, *QPM *Quality Protein Maize.

**Figure 2:**
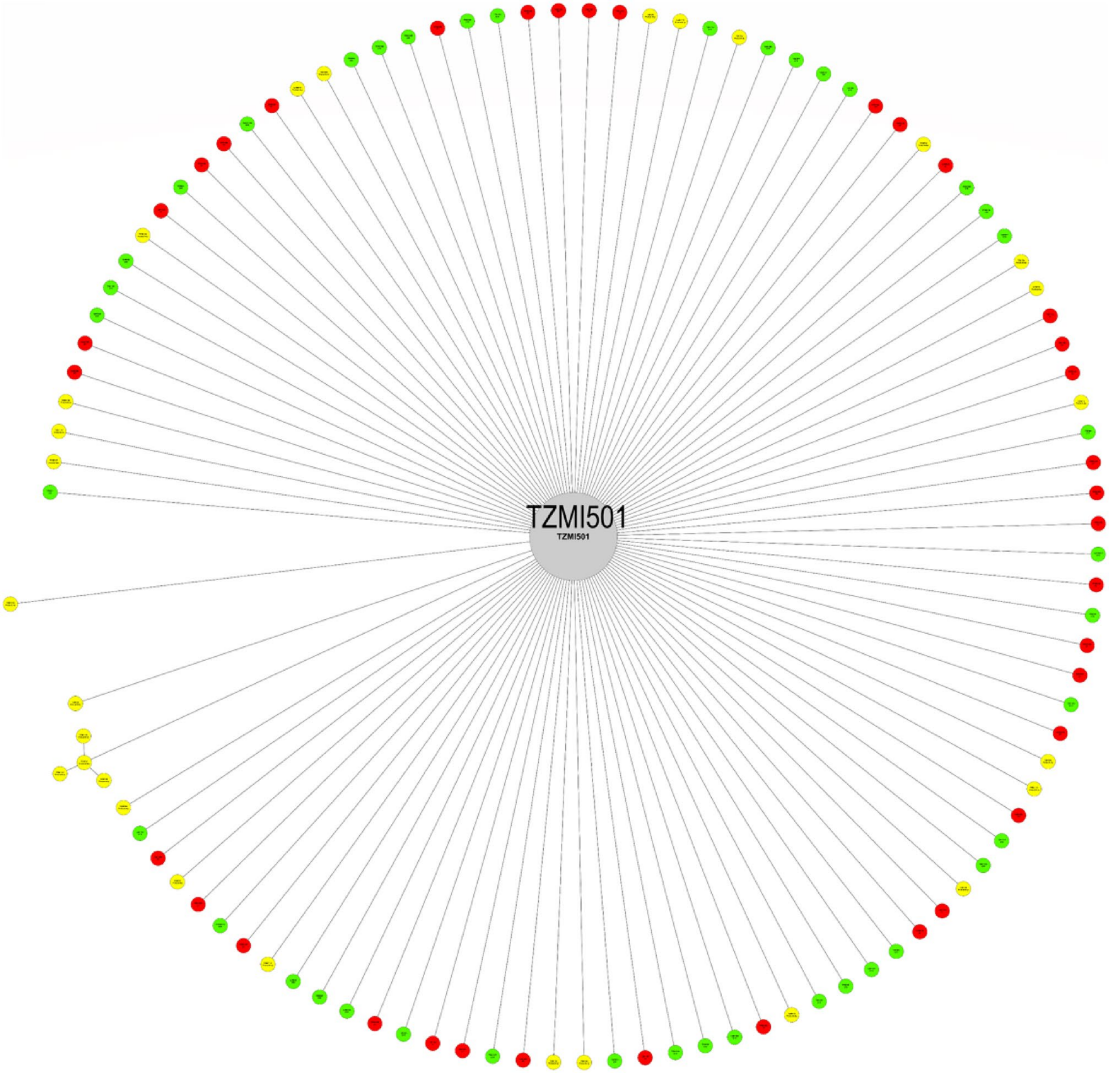
Key founder inbred lines identified through pedigree analysis. *PVA *Provitamin A, *STR Striga* resistance, *DT *Drought tolerant, *Early *Early maturing, *QPM *Quality Protein Maize.

The pedigree analysis identified 20 key founder inbred lines amongst the 88 elite inbred lines developed prior to 1995 that contributed to the development of at least one sub-tropical elite inbred line for drought tolerance (DT), earliness, PVA content, QPM, productivity, and resistance to *Striga* (STR). The founder line, TZMi501, representing our breeding program's flint heterotic group (HGA), was utilized in developing 101 elite DISTR inbred lines (Fig. 1). The pictorial pedigree relationship of TZMi501 with elite sub-tropical inbred lines is presented in Fig. 2. Also, two founder inbred lines, TZMi407-S and TZMi214, representing the dent heterotic group (HGB) in our breeding program, were involved in developing 97 and 79 elite inbred lines, respectively. TZMi407-S was primarily used to develop STR inbred lines, whereas TZMi214 was used principally to create PVA inbred lines (Fig. 1). The pictorial pedigree relationships of the two founder inbred lines with elite sub-tropical inbred lines are presented in Supplementary Figures S2 and S3. The pedigree-based relatedness of the remaining key founder inbred lines with the sub-tropical elite maize inbred lines could be visualized in Helium software version 1.19.09.03 by uploading the pedigree data provided in Supplementary Table S3.

**Figure 3:**
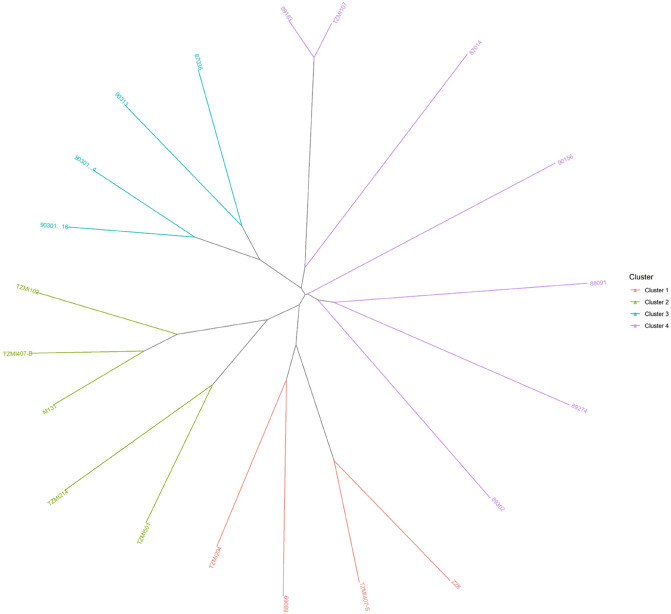
Neighbor joining tree based on Ward’s genetic distances among the 20 key founder inbred lines.

The SNP markers-based similarity matrix between the 20 key founder inbred lines, and the sub-tropical inbred lines is presented in Supplementary Table S4. TZMi501 was the key founder line having higher SNP-based similarity with elite sub-tropical inbred lines belonging to HGA in three major trait-based groups, including D|T, productivity, and STR lines (Supplementary Table S1). This founder line had average similarity scores varying from 67 to 72%, with inbred lines belonging to each trait group. The average similarity scores of the second-best founder line, TZMi407-S, ranged from 67 to 71%, with inbred lines representing the HGB of each of the same three major trait groups (Supplementary Table S1; Supplementary Table S4). Likewise, the third-best founder line, TZMi214, had average similarity scores ranging from 68 to 71%, with inbred lines present in each PVA trait group (Supplementary Table S1; Supplementary Table S4). Further analysis of the genetic similarity among the founder inbred lines is presented in Supplementary Table S4. The founder line TZMi501 had similar scores ranging from 66 to 81% with other founder inbred lines (Supplementary Table S4). As shown in Fig. 3, the clustering of the 20 founder inbred lines based on the neighbor-joining tree of genetic distance estimates revealed four clusters. Interestingly, the key founder inbred lines TZMi501, TZMi407-S, and TZMi214 were assigned to the same cluster. However, they represent two heterotic groups in our breeding program, possibly because they all share the same source of resistance (TZSR) to the maize streak virus disease used in IITA.

### SNP-based genetic diversity analyses among and within groups and among inbred lines

The 5032 SNPs data set recorded for the 623 elite sub-tropical maize inbred lines had a MAF ranging from 0.50 to 0.95 with an average of 0.77 (Table 2). The PIC values varied from 0.09 to 0.38, with a mean value of 0.26 in the entire panel. Gene diversity among the inbred lines varied from 0.10 to 0.50, with 36% of the loci lying between 0.10 and 0.24. Amongst these, 64% had gene diversity ranging from 0.25 to 0.50, with an average of 0.32 (Table 2 and Supplementary Table S5), indicating a high genetic diversity among the inbred lines. The level of heterozygosity also varied from 0.003 to 0.91, with an average of 0.08. About 96% of the markers had heterozygosity values ranging from 0.003 to 0.25 (Supplementary Table S5).

**Table 2:** Summary statistics for 5032 polymorphic SNP markers.

Summary	Average	Minimum	Maximum
Major allele frequency	0.77	0.50	0.95
Gene diversity	0.32	0.10	0.50
Heterozygosity	0.08	0.003	0.91
Polymorphic information content	0.26	0.09	0.38

**Figure 4:**
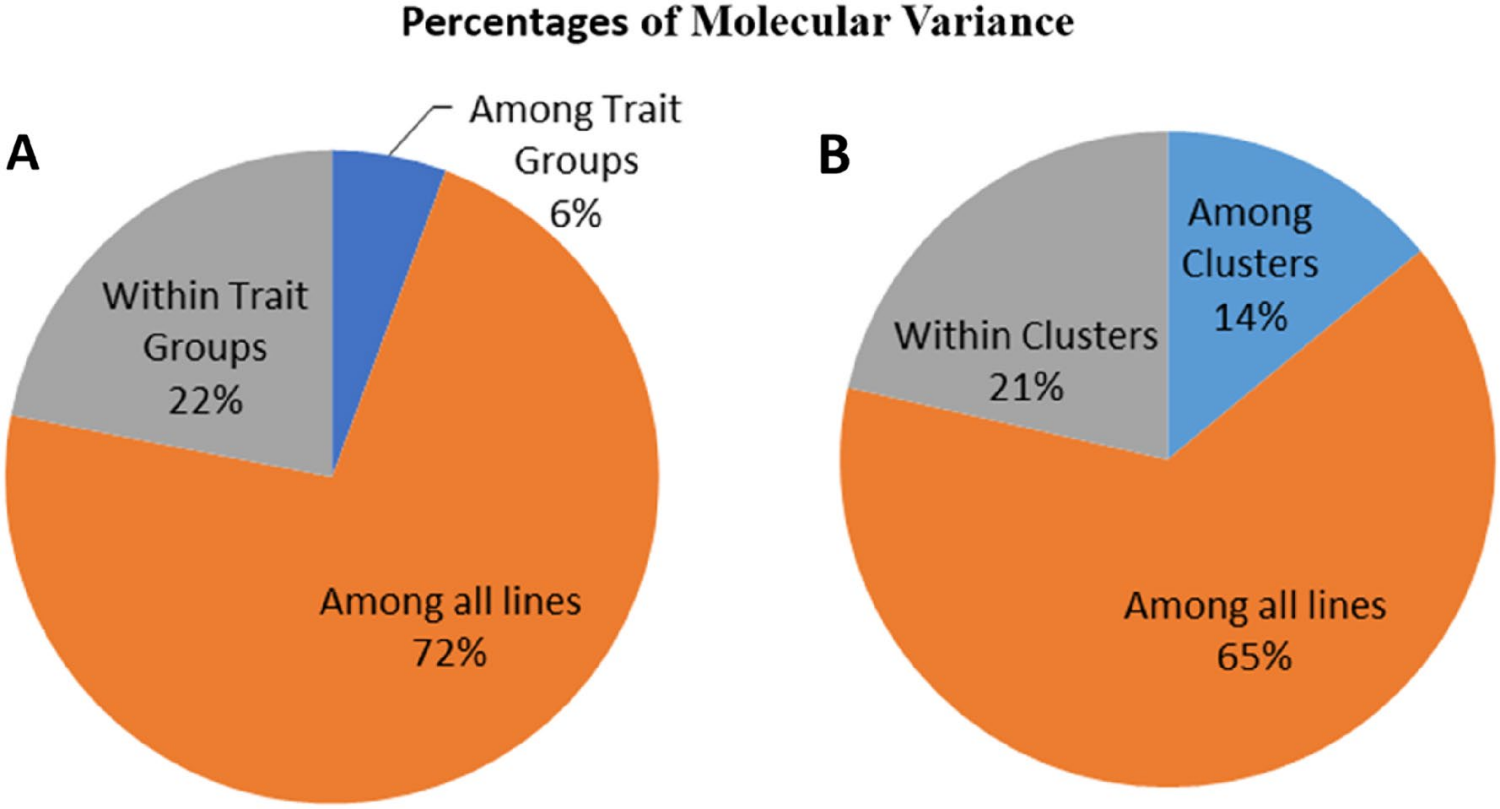
(**A**) Phenotypic trait-based percentage of molecular variance. (**B**) Genetic Structure-based percentage of molecular variance (AMOVA).

An analysis of molecular variance (AMOVA) was conducted to assess the relatedness among trait-based line subgroups as well as the founder line group to determine the level of genetic differentiation among subgroups. The AMOVA showed significant differentiation (*P* = 0.001) among trait-based groups, accounting for only 6% of the molecular variance. The respective variations among inbred lines within trait-based groups and among all inbred lines represent 22.72% of the total molecular variance (Fig. 4). Further analyses of the pairwise fixation index^24^ showed that the founder inbred lines had different levels of relatedness with other trait-based groups, with the founder inbred lines being more closely related to the group of productive inbred lines (Table 3). The founder inbred lines exhibited the highest genetic differentiation with the PVA inbred lines (*F*st = 0.114). Also, the PVA inbred lines had high genetic differentiation (0.10 to 0.12), with inbred lines belonging to other trait-based groups. In contrast, the productive inbred lines had the lowest fixation index values with other trait-based groups.

**Table 3:** Pairwise Fst values between the subgroups of inbred lines.

	DT	EARLY	Founder	Productive	PVA	QPM	STR
DT (N = 89)	0.000						
EARLY (N = 11)	0.076	0.000					
Founder (N = 20)	0.039	0.067	0.000				
Productive (N = 192)	0.022	0.067	0.015	0.000			
PVA (N = 86)	0.116	0.126	0.114	0.101	0.000		
QPM (N = 31)	0.051	0.087	0.051	0.035	0.124	0.000	
STR (N = 194)	0.033	0.076	0.027	0.023	0.111	0.057	0.000

### Population structure analysis

**Figure 5:**
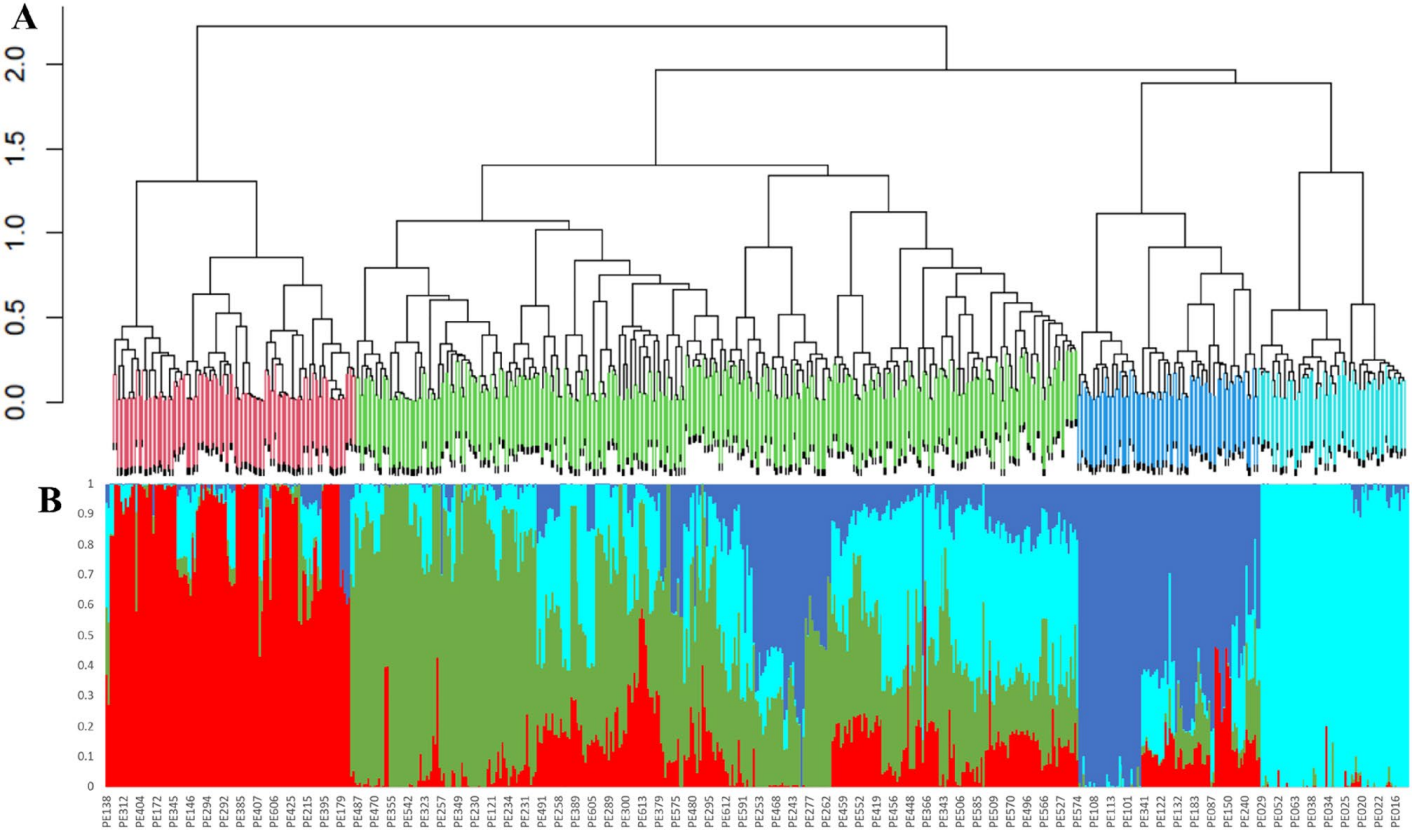
(**A**) Clustering of 623 sub-tropical maize inbred lines using Ward’s method; (**B**) Estimated population structure of the inbred lines as revealed by the 5032 SNP markers for K = 4. Cluster-1, Cluster-2, Cluster-3, and Cluster-4 are coloured with red, green, blue, and aqua blue colour, respectively.

Ward’s hierarchical cluster analysis separated the inbred lines into four clusters (Fig. 5A). Cluster-1 consisted of 117 (18.8%) inbred lines; cluster-2 contained 348 (55.8%) inbred lines, including 18 founder inbred lines; cluster-3 was composed of 87 (13.9%) inbred lines, including two founder inbred lines, while Cluster-4 comprised 71 (11.4%) inbred lines. The inbred lines belonging to the DT trait group were assigned to Cluster-1 (15%), Cluster-2 (80), and Cluster-3, with each cluster having a mixture of HGA and HGB inbred lines assigned by breeders (Supplementary Table S6). All early inbred lines assigned to HGB by breeders were included in Cluster-2. All the founder lines but two were also assigned to Cluster-2. The founder lines represent the two heterotic groups (HGA and HGB) assigned by the breeders (Supplementary Table S6). Amongst the productive trait group, 22% was allocated to Cluster-1, and 70% was assigned to Cluster-2, with the remaining 8% assigned to Cluster-3 and Cluster-4. Again, each cluster contains a mixture of HGA and HGB assigned by the breeders. Close to 80% of the PVA inbred lines were assigned to Cluster-4, with the remaining 20% assigned to the three clusters (Supplementary Table S6). The QPM lines belonging to the different heterotic groups assigned by breeders were divided into Cluster-1 (16%) and Cluster-2 (84%). Likewise, the STR inbred lines representing the other heterotic groups were assigned to Cluster-1 (27%), Cluster-2 (40%), and Cluster-3 (33) (Supplementary Table S6). Therefore, the SNP markers did not cluster the inbred lines along the heterotic groups assigned by breeders. Supplementary Table S6 Further analyses using AMOVA revealed significant differentiation (*P* = 0.001) among clusters accounting for only 14% of the molecular variance (Fig. 4). The variations among inbred lines within clusters and among all inbred lines represent 21% and 65% of the total molecular variance, respectively.

Analysis of the population structure among all sub-tropical elite maize inbred lines showed the largest delta K value at K = 4 (Supplementary Figure S4), suggesting that the inbred lines could be classified into 4 clusters (Fig. 5B). The structure analysis agreed with Ward’s hierarchical clustering in classifying the inbred lines into the same clusters. Only 22 inbred lines assigned to Ward’s Cluster-2 were classified into different structure-based clusters. Moreover, 245 inbred lines with membership probabilities below 60% were assigned to a mixed group. Among the 20 key founder inbred lines, 12 had membership probabilities below 60% and were assigned to the mixed group (Table 4). The remaining founder inbred lines were grouped in Cluster-2 and Cluster-3 (Table 4).

**Table 4:** Structure analysis-based ancestry, heterotic group, and target trait of 20 key founder inbred lines using 5032 SNP markers.

PEDNO	Founder	Cluster-1	Cluster-2	Cluster-3	Cluster-4	Genetic Structure	Ward's cluster	Heterotic group	Target trait
PE553	Z28	0	0	1	0	3	3	A	Productivity
PE554	87014	0.02	0.23	0.08	0.67	4	2	A	Productivity
PE555	87036	0.01	0.32	0.21	0.46	mixed	2	B	STR, Productivity
PE556	88069	0.01	0.21	0.77	0.01	3	2	A	PVA, Productivity
PE557	88091	0.08	0.23	0.2	0.5	mixed	2	A	STR
PE562	89183	0.15	0.25	0.15	0.45	mixed	2	A	Productivity
PE572	89274	0.15	0.15	0.21	0.49	mixed	2	B	STR, Productivity
PE576	89302	0.1	0.24	0.2	0.47	mixed	2	A	Productivity
PE582	90156	0.11	0.24	0.13	0.53	mixed	2	A	STR, Productivity
PE592	90301	0	0.33	0.3	0.37	mixed	2	B	STR, Productivity
PE593	90301	0.03	0.38	0.2	0.39	mixed	2	B	STR, Productivity
PE594	90313	0.08	0.34	0.16	0.43	mixed	2	A	STR
PE597	M131	0	1	0	0	2	2	A	STR, Productivity
PE601	TZMi102	0	0.83	0.09	0.08	2	2	A	STR, Productivity
PE604	TZMi107	0.14	0.25	0.16	0.45	mixed	2	A	Productivity
PE607	TZMi204	0.13	0.34	0.52	0.01	mixed	2	B	PVA, Productivity
PE615	TZMi214	0.56	0.38	0	0.06	mixed	2	B	PVA, Productivity
PE618	TZMi407	0	1	0	0	2	2	B	Productivity
PE619	TZMi407-S	0	0.07	0.93	0	3	3	B	DT, Productivity, STR
PE620	TZMi501	0.13	0.87	0	0	2	2	A	DT, Productivity, STR

*PVA *Provitamin A, *STR Striga* resistance, *DT *Drought tolerance, *Early *Early maturity, *QPM *Quality Protein Maize.

**Figure 6:**
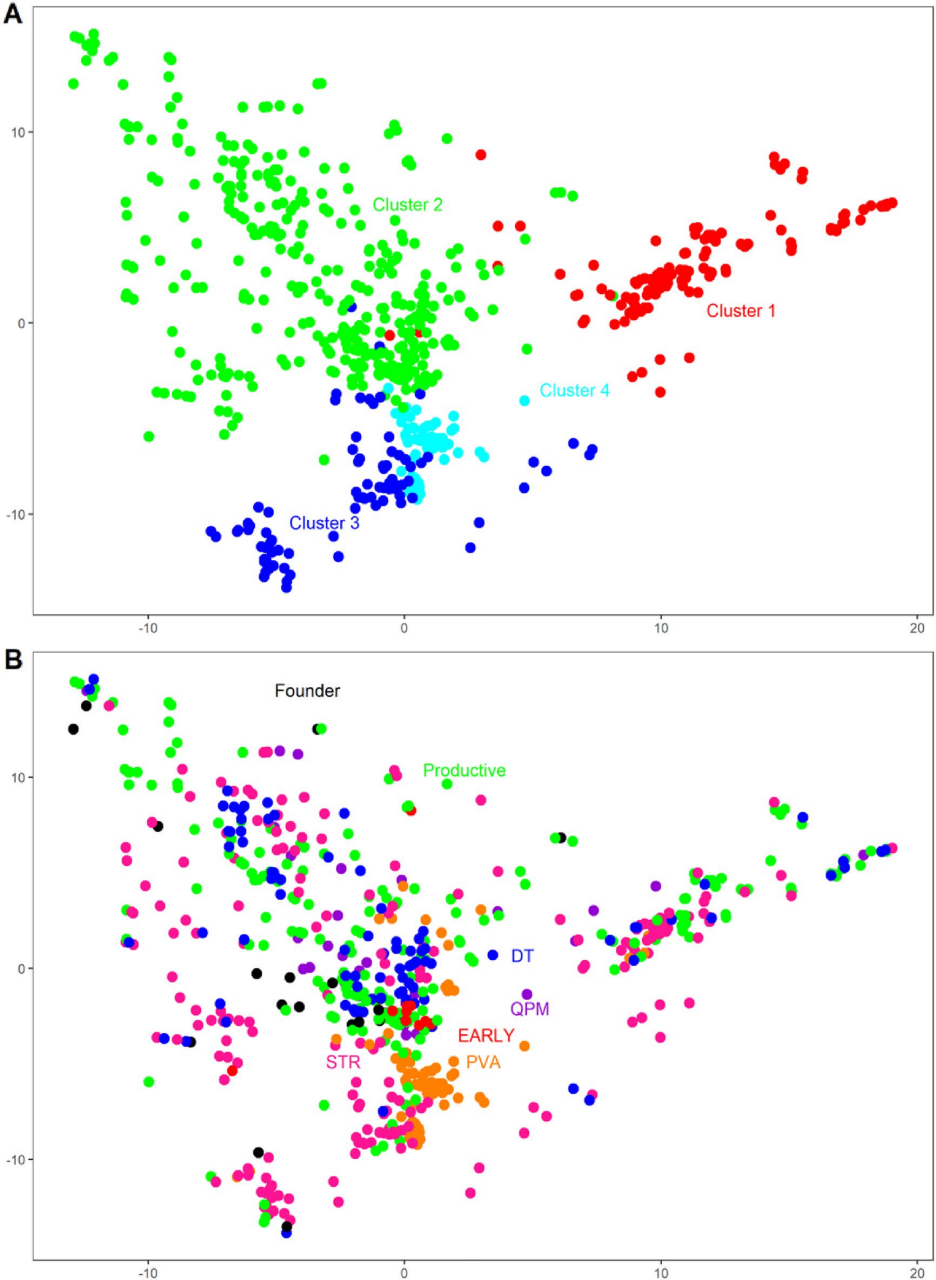
Principal Component Analysis (PCA) of the SNP data of the 623 sub-tropical maize inbred lines. The X and Y axes represent PC1 and PC2 respectively. Colours are assigned based on (**A**) Ward’s hierarchical cluster analyses (**B**) sub-groups of the inbred lines.

Principal component analysis (PCA) confirmed Ward’s hierarchical cluster analyses and the genetic structure by separating the sub-tropical maize inbred lines into four clusters (Fig. 6A). The classification of the inbred lines based on trait-based groups showed that the founder inbred lines were grouped close to the Productive, STR, and DT inbred lines (Fig. 5B). The EARLY and PVA inbred lines were grouped closely, whereas the Productive, STR, DT, and founder inbred lines were dispersed across different clusters (Fig. 6B).

## Discussion

The alarming population growth and climate change are grave challenges facing agricultural production globally^31^. Therefore, there is a pressing need to optimize the use of currently available genetic resources to improve grain yield and other agronomic and adaptive traits of the major crops, including maize. Over two decades, the maize improvement program at IITA has made significant progress in developing maize inbred lines with desirable agronomic features and adaptation to sub-tropical environments and shared with partners^32^. Different breeding methods were deployed to generate the inbred lines, including the introgression of desirable genes from exotic tropical and temperate inbred lines, followed by selection for improvements in target traits. Amongst the 88 elite sub-tropical inbred lines developed prior to 1995, 20 founder inbred lines were sources of adaptive traits in the subsequent development of the sub-tropical inbred lines with high grain yields, earliness, drought tolerance, *Striga* resistance, high lysine, tryptophan, and provitamin A content. In the present study, pedigree information was used to identify the founder inbred lines with significant contributions to the development of the different sets of inbred lines with improved target traits^12^. Such information is useful for breeders in IITA and partner institutions in their search for useful germplasm to further improve maize in sub-tropical environments. Molecular markers were also used to assess the genetic relationships between the founder inbred lines and the resultant trait-based groups of sub-tropical inbred lines^33^. Indeed, the genetic differentiation of trait-based groups of inbred lines from the founder inbred lines could provide insights for the optimal exploitation of elite inbred lines to develop superior hybrids to meet the current demand for higher yields, better nutrient content, and resistance to pests and diseases.

In the present study, founder inbred lines with significant contributions to the development of the sub-tropical maize inbred lines were identified based on pedigree information. The founder line, TZMi501 belonging to the flint heterotic group (HGA) in our breeding program, was the most valuable line contributing to the development of the highest number of inbred lines. This inbred line was used to develop mainly STR, DT, and productive inbred lines and thus could be used in other breeding programs for the improvement of these target traits. The founder line, TZMi4017-S, belonging to the dent heterotic group (HGB) in our program, was involved in developing many STR inbred lines, whereas TZMi214, which also belongs to the dent heterotic group (HGB), was involved in the development of many PVA inbred lines. TZMi214 could benefit breeding programs aiming at increasing PVA content in maize. Previous studies have used a similar approach to identify founder inbred lines with significant contributions to developing various inbred lines with United States plant variety protection certificates^10–13^.

Line TZMi501, identified as the top founder line based on pedigree information, was also the top founder line identified using the 5032 SNP-based genetic relationships with the sub-tropical inbred lines. As expected, TZMi501 had high genetic similarity with the DT (72%), STR (71%), and productive inbred lines (72%). Even though the pedigree analysis did not show any relationships between TZMi501 and the QPM inbred lines, this line had a high genetic similarity (72%) with QPM inbred lines. Such observation confirms the findings of Messmer et al.^34^ who reported that pedigree information could be incomplete and may not be useful in some cases. The founder inbred lines TZMi407-S, and TZMi214 had high genetic similarities with the STR (71%) and PVA (68%) inbred lines, respectively, consistent with the results from pedigree relationships. It should be noted that the 20 key founder inbred lines identified in the present study were separated into different clusters. The founder inbred lines belonging to the same clusters could be aligned to existing heterotic groups and used for broadening the genetic base of the breeding materials in other breeding programs targeting the improvement of the same set of traits.

The 5032 SNP markers found a significant level of genetic diversity among the sub-tropical inbred lines with an average GD of 0.32, which was comparable to the 0.39 GD value reported by^35^ for 527 inbred lines genotyped with Illumina GoldenGate assays. The PIC value of 0.26 obtained from this study was also slightly lower than the 0.28 value for 362 maize inbred lines genotyped with Illumina MaizeSNP50 SNP markers and 0.29 value for 103 inbred lines genotyped with LGC genomics SNP markers, reported by Zhang et al.^36^ and Abu et al.^37^. However, this value is higher than the 0.19 PIC value for 94 tropical maize inbred lines genotyped with Diversity Arrays Technology (DArT) SNP markers^38^.

The AMOVA showed some level of differentiation among the founder inbred lines and other trait-based groups. However, most of the diversity resided among inbred lines within trait-based groups and among inbred lines. The founder inbred lines were found to be more closely related to the group of productive inbred lines, possibly due to their extensive use for increasing grain yields and other adaptive traits. In contrast, the founder inbred lines showed greater genetic divergence from the PVA inbred lines because only a few founder inbred lines were used for developing the inbred lines. Moreover, desirable genes associated with PVA accumulation in maize were introgressed into the tropical inbred lines using temperate donor parents during the development of the PVA inbred lines, which were used as parents for generating the sub-tropical PVA inbred lines.

Ward’s hierarchical clustering, structure, and PCA analysis demonstrated the presence of similar genetically distinct groups of sub-tropical maize inbred lines used in our study. The productive, STR, DT, and founder lines assigned to existing HGA and HGB by breeders were scattered across different clusters, indicating that these inbred lines originated from crosses of diverse parents during their development. Some inbred lines are productive and *Striga* resistant, whereas others combine high yield potential with drought tolerance. Only the PVA inbred lines containing temperate germplasm in their genetic backgrounds were grouped into a major cluster because they originated from crosses of tropical PVA inbred lines with a few elite sub-tropical inbred lines. The lack of correspondence between the existing heterotic groups and the clusters generated using SNP markers could arise due to the origin of the parents of the sub-tropical inbred lines from broad-based populations and the germplasm of unknown heterotic patterns. The resulting cluster analyses and testcross evaluation could thus be used as the bases to refine further the heterotic patterns of the diverse sup-tropical maize inbred lines.

## Conclusion

This study elucidated the genetic relationships between key founder inbred lines and all elite sub-tropical maize inbred lines developed from them in the Maize Improvement program at IITA for two decades. The study has provided insight into the structure of the genetic diversity for the 623 sub-tropical maize inbred lines. Twenty key founder inbred lines with significant contributions to the development of DT, EARLY, Productive, STR, PVA, and QPM inbred lines were identified based on both pedigree data and SNP markers-based similarity estimates. TZMi501, TZMi407-S, and TZMi214 were identified as the most widely used founder inbred lines. TZMi501 was mainly used to develop DT, STR, and Productive inbred lines, whereas TZMi407-S and TZMi214 were used to develop the STR and the PVA inbred lines, respectively. Considerable genetic diversity was found among the sub-tropical inbred lines included in the present study. All the 623 inbred lines were consistently separated into four clusters based on Ward’s hierarchical clustering, structure, and PCA analyses. The productive, STR, DT, and founder inbred lines were scattered across different clusters, whereas the PVA inbred lines were grouped into the same cluster. The results of the analysis of AMOVA highlighted the presence of significant differences among trait-based groups and clusters and among inbred lines within the trait-based groups and clusters. These results will facilitate the selection of diverse inbred lines belonging to the different trait-based groups for use in breeding programs targeting the improvement of desirable traits in specific heterotic groups. The clusters also provide a useful breeding structure to generate inbred lines aligned to specific heterotic groups to exploit higher expression of heterosis in hybrids in IITA and partner breeding programs.

### Supplementary Information

Supplementary Figures.

Supplementary Table S1.

Supplementary Table S2.

Supplementary Table S3.

Supplementary Table S4.

Supplementary Table S5.

Supplementary Table S6.

## Data Availability

All data generated or analysed during this study are included in this published article (and its Supplementary Information files).The DArTseq datasets (Supplementary Table S2) used in the present study have also been deposited at the IITA repository (https://doi.org/10.25502/3qtm-7589/d). Link to CKAN: https://data.iita.org/dataset/data-for-tropical-mid-altitude-maize-inbred-lines-and-key-founder-lines-for-diversity-assessment.

## References

[1] Buddenhagen IW, Bosque-Perez N (1999). Historical overview of breeding for durable resistance to maize streak virus for tropical Africa. S. Afr. Tydskr. Plant Grond.

[2] IITA, *Sustainable food production in sub-Saharan Africa 1. IITA’s contributions*. Ibadan Nigeria (1992)

[3] Menkir A, Gedil M, Franco J (2010). Analysis of genetic structure and diversity among lowland and mid-altitude adapted maize inbred lines with AFLP markers. J. Food Agric. Environ..

[4] Everett L (1994). Registration of four tropical midaltitude maize germplasm populations. Crop Sci..

[5] Everett L (1994). Registration of 18 first-cycle tropical midaltitude maize germplasm lines. Crop Sci.

[6] Everett L (1994). Registration of 19 second cycle tropical midaltitude maize germplasm lines. Crop Sci.

[7] Kim, S.K. Achievements, challenges and future direction of hybrid maize research and production in West and Central Africa. in *Regional Maize Workshop*, (IITA, 1997).

[8] Technow F (2014). Identification of key ancestors of modern germplasm in a breeding program of maize. Theor. Appl. Genet..

[9] Smith JSC (2004). Changes in pedigree backgrounds of pioneer brand maize hybrids widely grown from 1930 to 1999. Crop Sci..

[10] Mikel MA (2008). Genetic diversity and improvement of contemporary proprietary North American dent corn. Crop Sci..

[11] Mikel MA (2011). Genetic composition of contemporary U.S. commercial dent corn germplasm. Crop Sci..

[12] Mikel MA, Dudley JW (2006). Evolution of north American dent corn from public to proprietary germplasm. Crop Sci..

[13] Smith S (2007). Pedigree background changes in U.S. hybrid maize between 1980 and 2004. Crop Sci..

[14] Coffman SM (2019). Haplotype structure in commercial maize breeding programs in relation to key founder lines. Theor. Appl. Genet..

[15] Minamikawa MF (2021). Tracing founder haplotypes of Japanese apple varieties: application in genomic prediction and genome-wide association study. Hortic. Res..

[16] Everett LA (1991). Midaltitude Maize Breeding: Populations, inbreds and hybrids from the IITA-NCRE-Cameroon Program.

[17] Kim SK (2003). Maize germplasm developed and studied by Dr. Soon-Kwon Kim and his colleagues from Africa, Asia, and USA. A joint publication of Kyungpook National University and International Corn Foundation.

[18] Zebire D (2021). Identifying suitable tester for evaluating Striga resistant lines using DArTseq markers and agronomic traits. PLoS ONE.

[19] Bradbury PJ (2007). TASSEL: Software for association mapping of complex traits in diverse samples. Bioinformatics.

[20] Shaw PD (2014). Helium: Visualization of large scale plant pedigrees. BMC Bioinf..

[21] Milne I (2010). Flapjack—graphical genotype visualization. Bioinformatics.

[22] Liu K, Muse SV (2005). PowerMarker: an integrated analysis environment for genetic marker analysis. Bioinformatics.

[23] Peakall R, Smouse PE (2012). GenAlEx 6.5: genetic analysis in excel. Population genetic software for teaching and research–an update. Bioinformatics.

[24] Hufstetler EV (2007). Genotypic variation for three physiological traits affecting drought tolerance in soybean. Crop Sci..

[25] Holsinger KE, Weir BS (2009). Genetics in geographically structured populations: Defining, estimating and interpreting F_ST_. Nat. Rev. Genet..

[26] Pritchard JK, Stephens M, Donnelly P (2000). Inference of population structure using multilocus genotype data. Genetics.

[27] Evanno G, Regnaut S, Goudet J (2005). Detecting the number of clusters of individuals using the software structure: A simulation study. Mol. Ecol..

[28] Alexander DH, Novembre J, Lange K (2009). Fast model-based estimation of ancestry in unrelated individuals. Genome Res..

[29] Paradis E, Claude J, Strimmer K (2004). APE: Analyses of phylogenetics and evolution in R language. Bioinformatics.

[30] R Core Team (2021). R: A Language and Environment for Statistical Computing.

[31] Romay MC (2013). Comprehensive genotyping of the USA national maize inbred seed bank. Genome Biol..

[32] Gedil M, Menkir A (2019). An integrated molecular and conventional breeding scheme for enhancing genetic gain in maize in Africa. Front. Plant Sci..

[33] Bernardo R (2000). Parental contribution and coefficient of coancestry among maize inbreds: Pedigree, RFLP, and SSR data. Theor. Appl. Genet..

[34] Messmer MM (1993). Relationships among early European maize Inbreds: II. Comparison of pedigree and RFLP data. Crop Sci..

[35] Yang X (2011). Characterization of a global germplasm collection and its potential utilization for analysis of complex quantitative traits in maize. Mol. Breeding.

[36] Zhang X (2016). Characterizing the population structure and genetic diversity of maize breeding germplasm in Southwest China using genome-wide SNP markers. BMC Genomics.

[37] Abu P (2021). Genetic diversity and inter-trait relationship of tropical extra-early maturing quality protein maize inbred lines under low soil nitrogen stress. PLoS ONE.

[38] Adu GB (2019). Genetic diversity and population structure of early-maturing tropical maize inbred lines using SNP markers. PLoS ONE.

