# Author Correction: Genetic structure analysis and identifying key founder inbred lines in diverse elite sub-tropical maize inbred lines

**DOI:** 10.1038/s41598-023-40386-0

**Published:** 2023-08-15

**Authors:** Melaku Gedil, Abdoul‑Raouf Sayadi Maazou, Degife A. Zebire, Ana Luísa Garcia‑Oliveira, Nnanna Unachukwu, César Petroli, Sarah Hearne, Leslie A. Everett, Soon‑Kwon Kim, Abebe Menkir

**Affiliations:** 1 International Institute of Tropical Agriculture, PMB 5320, Ibadan, 200001 Nigeria; 2 Kindo Seeds, Niamey, Niger; 3 Department of Plant Science, College of Agricultural Sciences, Arba Minch University, Arba Minch, Ethiopia; 4 International Maize and Wheat Improvement Center, Excellence in Breeding, Ibadan, Nigeria; 5 International Maize and Wheat Improvement Center, Carretera México‑Veracruz Km. 45 El Batán, C.P. 56237 Texcoco, Mexico; 6 International Maize and Wheat Improvement Center (CIMMYT), ICRAF House, UN Avenue, P.O. Box 1041-00621, Nairobi, Kenya; 7 Department of Molecular Biology, College of Biotechnology, CCS Haryana Agricultural University, Hisar, Haryana 125004 India; 8 Department of Agronomy and Plant Genetics, University of Minnesota, 411 Borlaug Hall, 1991 Upper Buford Circle, Saint Paul, MN 55108 USA; 9 Handong Global University, Pohang, Republic of Korea; 10 International Corn Foundation, Pohang, Republic of Korea

Correction to: *Scientific Reports* 10.1038/s41598-023-38980-3, published online 20 July 2023

The original version of this Article contained errors in affiliations 6, 7, 9 and 10. The correct affiliations are listed below.

6 International Maize and Wheat Improvement Center (CIMMYT), ICRAF House, UN Avenue, Nairobi, P.O. Box 1041-00621, Kenya.

7 Department of Molecular Biology, College of Biotechnology, CCS Haryana Agricultural University, Hisar 125004, Haryana, India.

9 Handong Global University, Pohang, Republic of Korea.

10 International Corn Foundation, Pohang, Republic of Korea.

The original Article has been corrected.

