# Supplementary material for: Genetic structure analysis and identifying key founder inbred lines in diverse elite sub-tropical maize inbred lines
Source: Sci Rep. 2023 Jul 20;13:11695. doi: 10.1038/s41598-023-38980-3 (PMC10359401; doi:10.1038/s41598-023-38980-3)
Supplement: Supplementary file 1 — Supplementary Figures. [file 41598_2023_38980_MOESM1_ESM.docx]

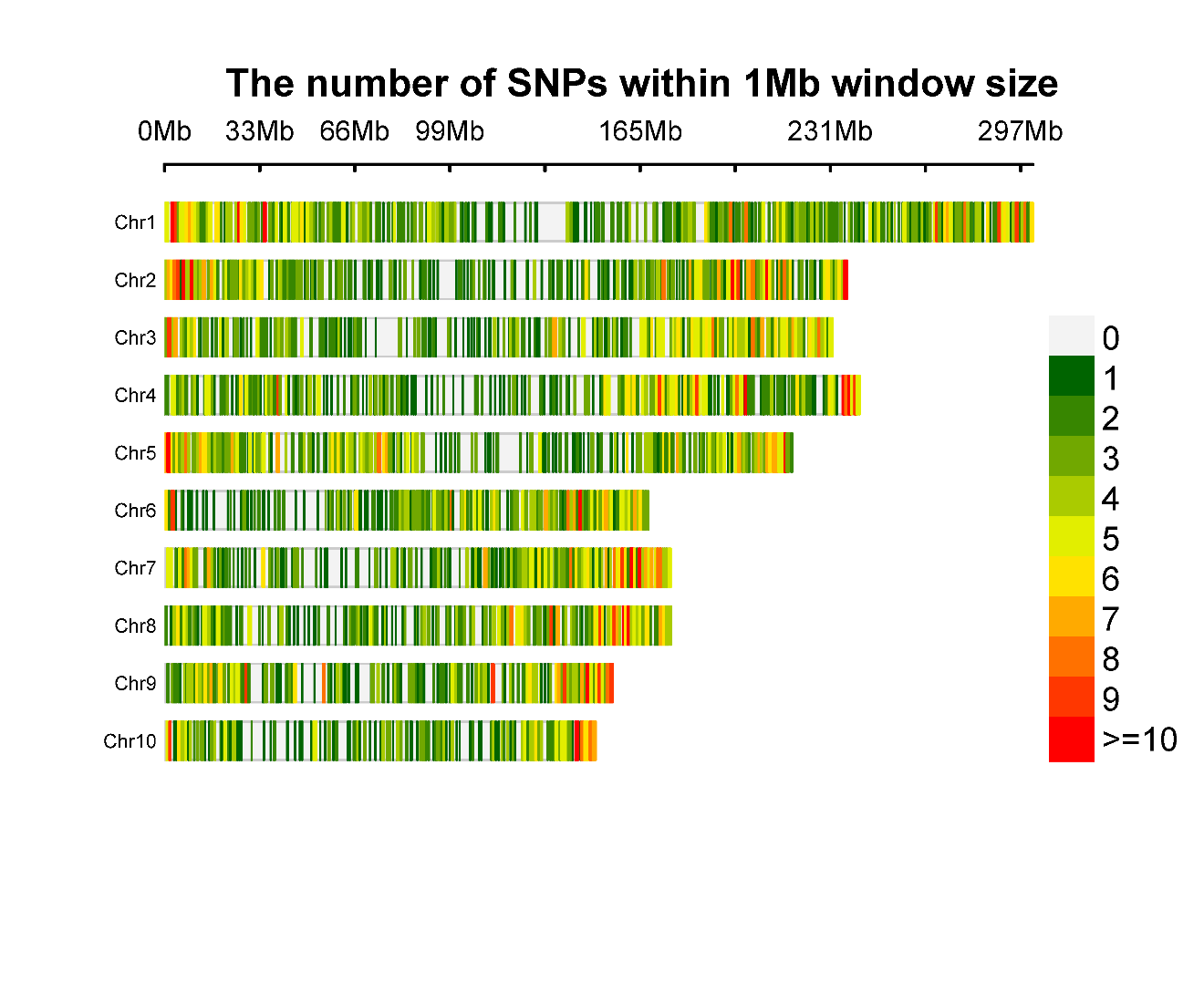


Supplementary Figure S1 Distribution of the SNP markers across the maize chromosomes


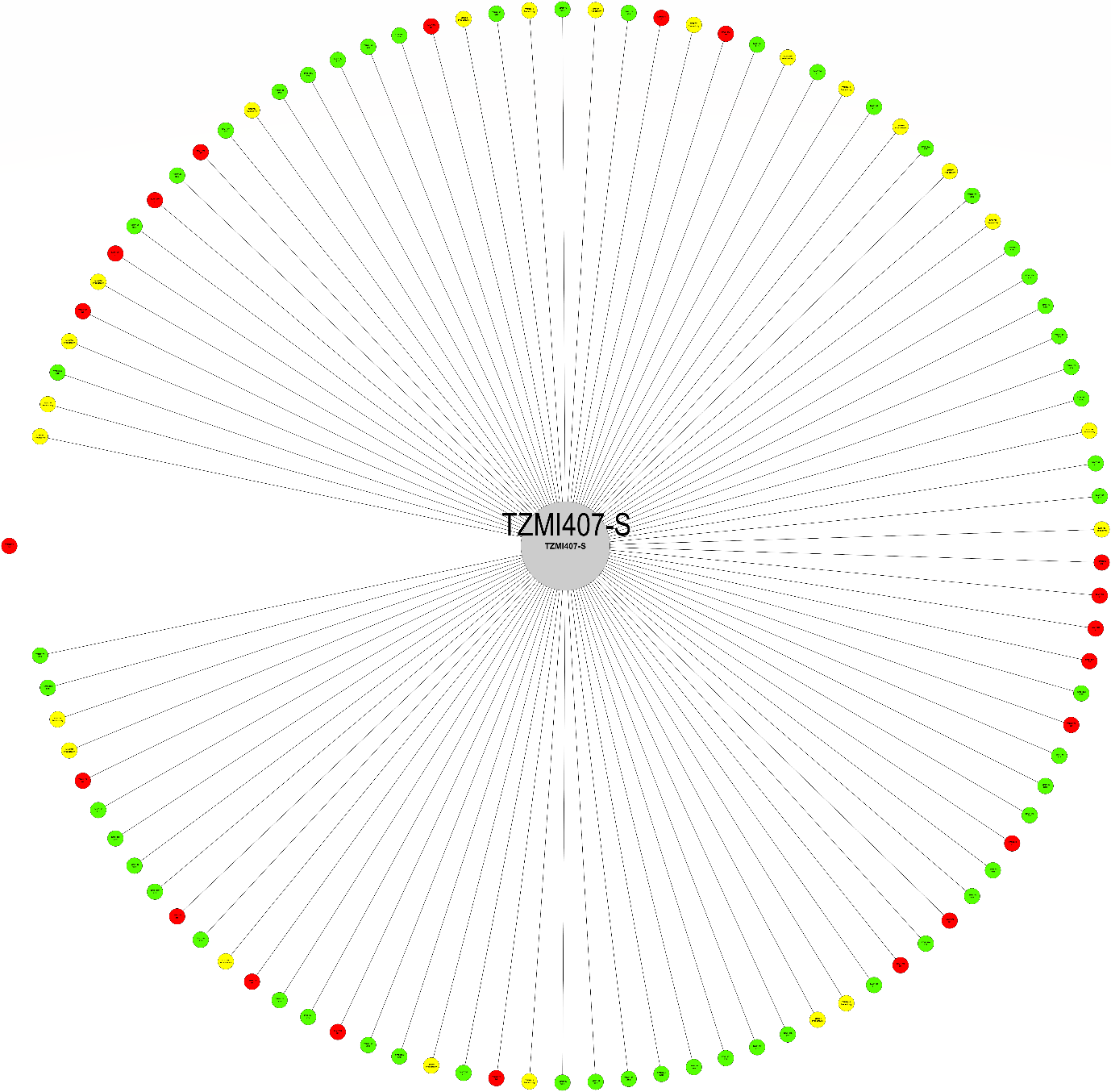


Supplementary Figure S2 Key founder line TZMI407-S identified through pedigree analysis. PVA = Provitamin A, STR = Striga resistance, DT = Drought tolerant, Early = Early maturing, QPM = Quality Protein Maize


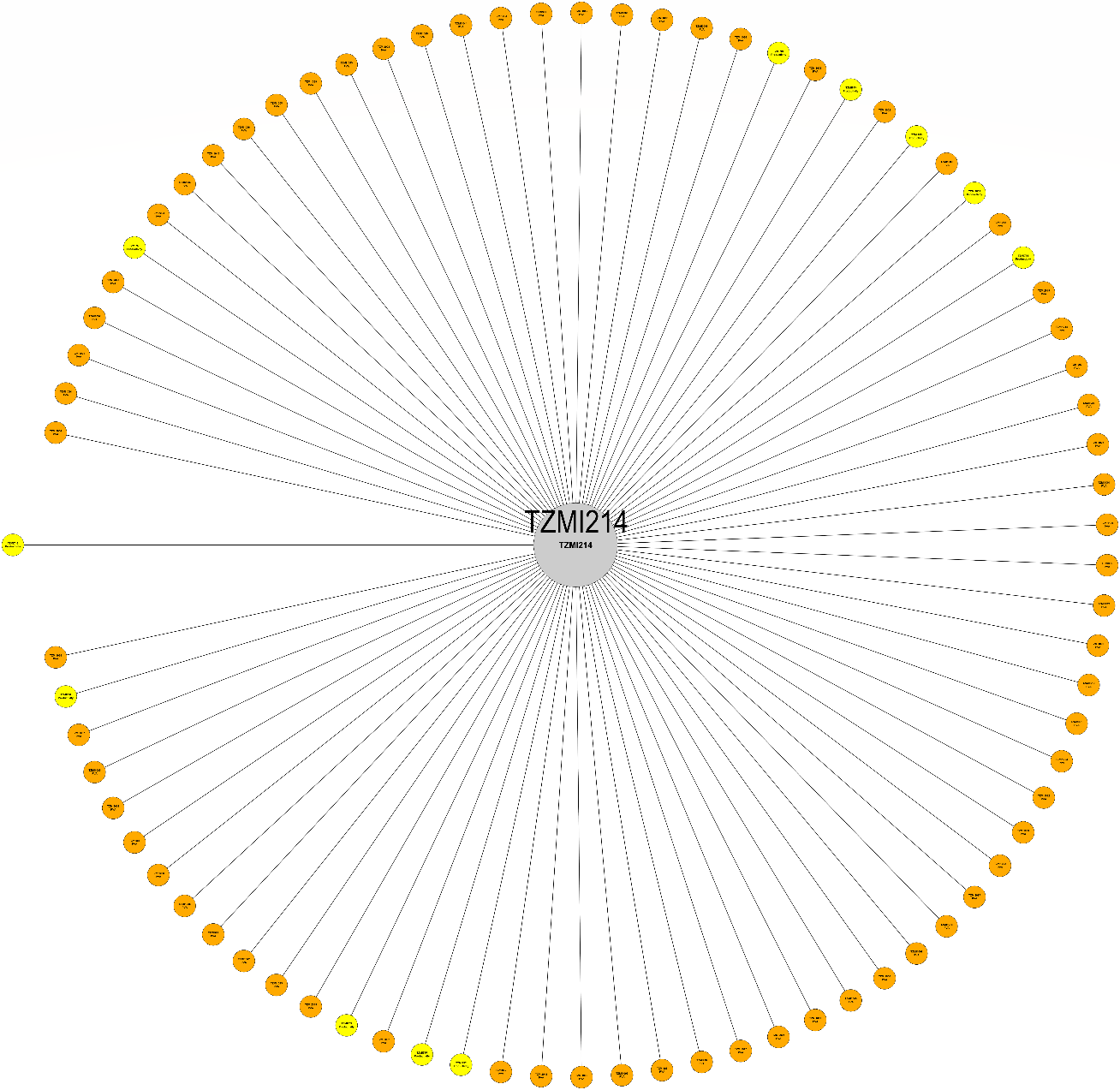


Supplementary Figure S3 Key founder line TZMI214 identified through pedigree analysis. PVA = Provitamin A


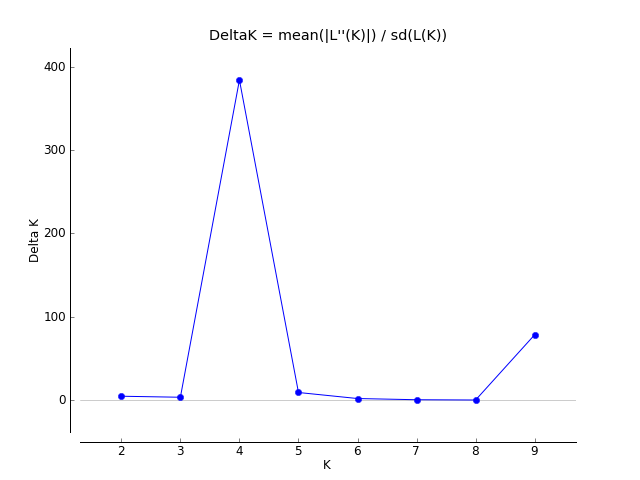


Supplementary Figure S4 Determination of the most appropriate K-value in structure analysis using Evanno’s Delta K.
